# Atypical Liver Ultrasound Image in a Boy with Autosomal Recessive Polycystic Kidney Disease (ARPKD) and New PKD1 Variant—A Case Report

**DOI:** 10.3390/genes16111244

**Published:** 2025-10-22

**Authors:** Agnieszka Turczyn, Grażyna Krzemień, Dominik Nguyen

**Affiliations:** 1 Department of Pediatrics and Nephrology, Medical University of Warsaw, 02-091 Warsaw, Poland; grazyna.krzemien@wum.edu.pl; 2 Department of Pediatric Radiology, Medical University of Warsaw, 02-091 Warsaw, Poland; dominik.nguyen@op.pl

**Keywords:** autosomal recessive polycystic kidney disease (ARPKD), polycystic kidney disease, PKHD1 gene, polycystic liver disease, liver cysts

## Abstract

**Background**: Autosomal recessive polycystic kidney disease (ARPKD) is a rare form of PKD that leads to the development of multiple renal cysts and hepatic fibrosis. **Aim**: The first documented case of large hepatic cyst associated with dual *PKHD1-PKD1* variants. **Case report**: We present the case of a 5-year-old boy with a kidney US image typical of ARPKD and numerous large cysts in the liver not typical for this disease. Genetic analysis revealed heterozygous missense mutations in the *PKHD1* gene (maternally, c.107C>T/p.Thr36Met in exon 3; paternally, c.4870C>T/p.Arg1624Rrp in exon 32) and an additional new variant in *PKD1* (maternally, c.5323G>A/p.Gly1775Ser in exon 32). Genetic tests excluded mutations in genes responsible for polycystic liver disease (PCLD). However, the presence of the *PKD1* mutation is clinically not clear due to the normal abdominal US image in the mother; it seems to be the most likely explanation for unusual phenotype in our patient. **Conclusions**: This case may contribute to the understanding of the phenotypic variability in ARPKD and the potential modifying role of mutations in other PKD-related genes. Comprehensive genetic panels are crucial for explaining atypical phenotypes and prognosis in patients with PKD.

## 1. Introduction

Autosomal recessive polycystic kidney disease (ARPKD, ORPHA:731, OMIM#263200) is a rare form of PKD with a prevalence of 1:20,000 live births in Europe, 1:26,500 in North, Central, and South America, and 1:8000 in an isolated population. The majority of ARPKD cases result in Polycystic Kidney and Hepatic Disease 1 (*PKHD1*) mutations, unique for each person. A small number of individuals reveal mutations in the DAZ interacting zinc finger protein 1 (*DZIP1L*) [1]. ARPKD is characterized by the development of multiple fluid-filled cysts in the renal parenchyma that enlarge over time and hepatic fibrosis. The formation of the cysts progressively impairs renal function and leads to chronic kidney disease (CKD). The presence of numerous large cysts in the liver is not typical for ARPKD. Coexistence of *PKHD1* and *PKD1* mutations may lead to an unusual phenotype of ARPKD [2,3]. Experimental studies indicated that the coexistence of mutations in *Pkhd1* and *Pkd1/Pkd2* is associated with a more rapid and severe manifestation of PKD [4].

According to our best knowledge, this is the first documented case of large hepatic cyst associated with dual *PKHD1-PKD1* variants.

## 2. Case Report

We report a case of a second pregnancy, male, first delivery, born naturally at 37 weeks of gestation, with birth weight 2700 g, Apgar score 9. The adaptation period was normal. The prenatal ultrasound (US) showed bilateral renal enlargement and borderline amniotic fluid volume in the second trimester. The postnatal US showed enlarged kidneys (67 mm in length, z-score of 3.27) with increased echogenicity, loss of corticomedullary differentiation, and multiple cysts, as well as liver hyperechogenic, scattered dilated hepatic triads (bile ducts), and hypoechogenic areas of irregular shape (possible cysts) in liver parenchyma. The blood pressure (BP) was normal; laboratory tests, besides hyperbilirubinemia (18.8 mg/dL) with the domination of conjugated bilirubin and increased activity of GGTP (439 U/L), were also normal. Family history revealed a normal abdominal US of the mother (at the age of 37 years). The father was diagnosed with hypertension (HT) during childhood and refused to undergo the US.

**Figure 1:**
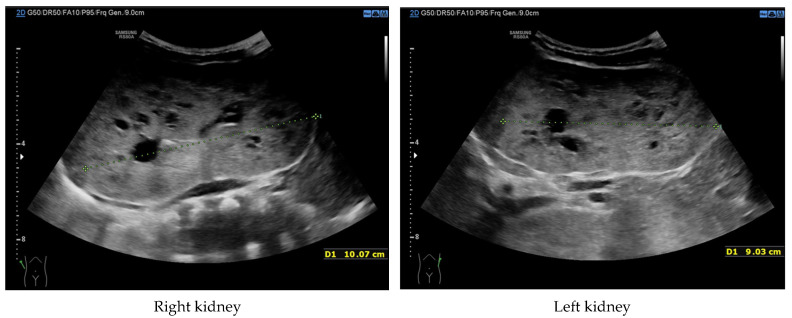
Ultrasonography of kidneys at the age of 4 months (enlarged kidneys with multiple cysts).

**Figure 2:**
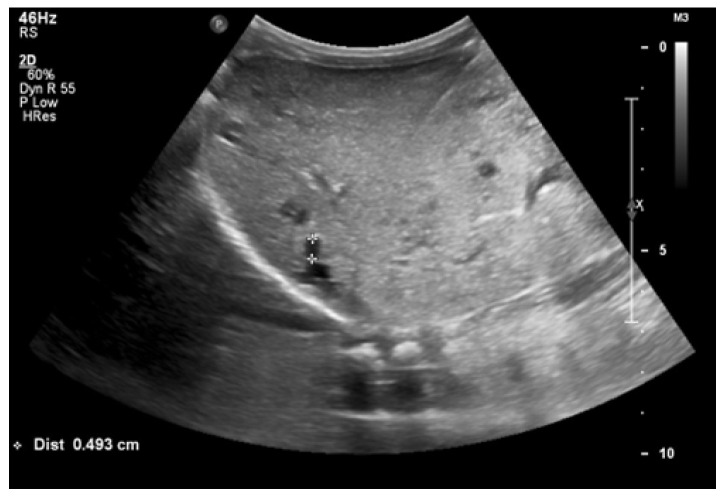
Ultrasonography of the liver at the age of four months (hyperechogenic, enlarged liver with multiple cysts).

At 4 months of age, the boy was admitted to the hospital due to a hypertensive crisis (BP 162/128 mmHg). Chest X-ray demonstrated cardiomegaly, and echocardiography (ECHO) showed a significant left ventricular hypertrophy and impaired contractility. Abdominal US detected increased kidneys length (right: 101 mm (z-score 8.72); left: 90 mm (z-score 6.59)), with increased echogenicity and loss of corticomedullary differentiation, as well as numerous cysts 5–6 mm in diameter. The liver was enlarged with increased echogenicity with cysts 6–8 mm in diameter (Figure 1 and Figure 2). Doppler US demonstrated high-resistance intrarenal blood flow (RI: 0.75–0.84). Laboratory tests showed microcytic anemia, renal function was assessed as serum creatinine, the glomerular filtration rate (GFR) was estimated according to the Schwartz formula, and liver function was all normal. The boy received multi-drug therapy to control hypertension and heart failure. ECHO, performed after 6 months, was correct.

**Figure 3:**
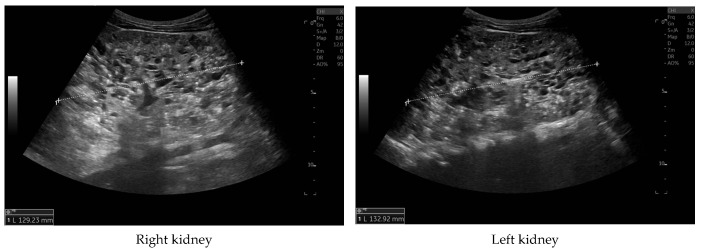
Ultrasonography of kidneys at the age of five years.

**Figure 4:**
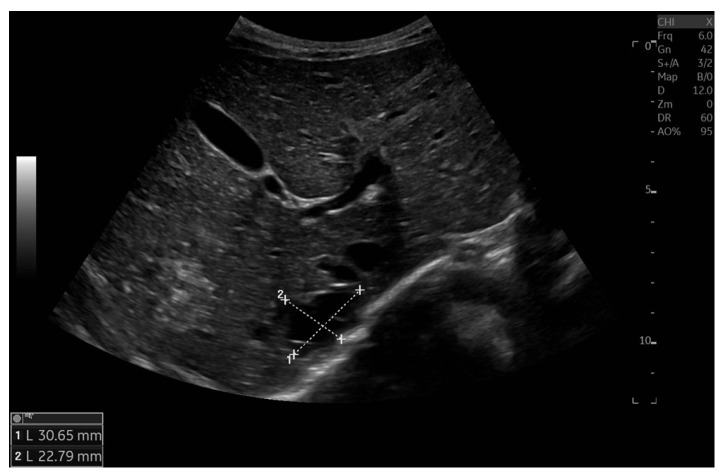
Ultrasonography of the liver with the largest cluster of cysts.

Currently, the boy is 5 years old. The US is similar to the previous one: elevated kidneys length (right: 130 mm (z-score 6.19); left: 133 mm (z-score 6.50)), with increased echogenicity, blurred corticomedullary differentiation, and multiple cysts with a maximum diameter up to 13–15 mm. The liver has a normal size, with numerous cysts up to 16 mm in diameter, with the largest cluster of cysts measuring 43 × 23 × 31 mm (Figure 3 and Figure 4). Laboratory tests show increased creatinine 0.79 mg/dL, urea 69,6 mg/dL, cystatin C 1.71 mg/L, uric acid 6.9 mg/dL, urine albumin to creatine ratio (ACR) 118.6 mg/g, and decreased GFR 55 mL/min/1.73 m^2^—stage III CKD. Other tests, including electrolytes, liver function, and urinalysis, were normal. The boy demands antihypertensive multi-drug therapy: angiotensin-converting enzyme inhibitors (ACEIs), calcium channel blockers, and beta-blockers.

Genetic diagnostics were carried out with the use of Next-Generation Sequencing (NGS). The panel of genes included PKD-related genes such as *DNAJB11*, *DZIPL1L*, *GANAB*, *HNF1B*, *PKD1*, *PKD2*, *PKHD1*, *NOTCH2*, *PAX2*, *UMOD*, *BICC1*, and *PRKCSH*, *LRP5*, *SEC63*, and *GANAB*-related polycystic liver disease (PCLD). The genetic tests revealed that the boy is a heterozygous carrier of two missense molecular variants in the *PKHD1* gene and new missense molecular variant in the *PKD 1* gene. The first variant in the *PKHD1* gene located on exon 3 demonstrated nucleotide record c.107C>T and protein record p.(Thr36Met), the second variant located on exon 32 demonstrated nucleotide record c.4870C>T and protein record p.(Arg1624Rrp). Both variants are known and described as pathogenic. Pedigree analysis carried out in the patient’s family showed the biparental origin of the mutation, c.107C>T from the mother and c.4870C>T from the father. The additional variant in the *PKD1* gene located on exon 32 gene showed nucleotide record c.5323G>A and protein record p.(Gly1775Ser). Carrier status analysis of the above variant showed that it was inherited from the proband’s mother. The genetic tests excluded mutations in genes responsible for PCLD.

## 3. Discussion

ARPKD is a severe form of PKD, with a mortality rate of up to 30–40% cases in the first year of life [5]. Approximately 80% of patient with ARPKD are diagnosed with hypertension (HT) within the first few months of life [6], and about 50% of patients progress to end-stage renal disease in the first decade of life [2]. Some cases are clinically silent until adulthood, with domination of liver symptoms [7].

The clinical diagnosis of ARPKD is suspected according to the modified Zerres criteria [8], which include typical for ARPKD findings in US: increased renal size, increased renal echogenicity, loss of corticomedullary differentiation, and one or more of the following: (1) lack of renal cysts in both parents in US, (2) typical liver imaging, clinical or laboratory confirmation of congenital hepatic fibrosis, (3) hepatic pathology showing characteristic ductal plate malformation, and (4) diagnosis of ARPKD in affected siblings. In many cases, genetic testing clearly confirms the diagnosis of the disease.

In our patient, antenatal suspicion of PKD was established in the second trimester of pregnancy based on enlarged kidneys and amniotic fluid limit values. The postnatal US showed typical for ARPKDkidney enlargement with heterogeneous parenchymal echogenicity, poor corticomedullary differentiation, and microcysts. Liver imaging revealed numerous large cysts. No fibrosis or other abnormalities were found in the liver. Although laboratory parameters assessing liver function are normal, liver fibrosis and laboratory signs of liver damage may appear at a later age [1]. HT was recognized at the age of 4 months. Rapid progression of CKD may indicate the need for starting renal replacement therapy in the next few years.

Liver involvement is present in all ARPKD patients. However, it consists of hepatic fibrosis, which leads to portal hypertension, variceal bleeding, esophageal varices, cholangitis, and/or nonobstructive dilatation of intrahepatic bile ducts, with the development of Caroli’s syndrome in some cases [5,9]. The presence of multiple large cysts in the liver is not typical for ARPKD, although a subset of individuals who carry the PKHD1 mutation develop small focal liver cysts [10].

The differential clinical and genetical diagnosis in children with ARPKD suspicion must include diseases that can imitate the ARPKD phenotype, like very early onset of autosomal dominant polycystic kidney disease (VEO-ADPKD), hepatocyte nuclear factor 1β (HNF1B) nephropathy, or nephronophthisis (NPHP). VEO-ADPKD is diagnosed during pregnancy or at the age of <18 months. The similarity between these two diseases includes enlarged hyperechogenic kidneys, oligohydramnios in very severe cases of ADPKD, early onset of HT, and progression to renal failure. The differences concern the image of the liver: significant liver fibrosis characterizes ARPKD, and the presence of liver cysts is typical in ADPKD patients [1,11]. The primary cause of hyperechogenic fetal kidneys is related to mutation of the transcription factor hepatocyte nuclear factor 1β (HNF1B). Nephropathy HNF1B belongs to autosomal dominant tubulointerstitial kidney disease (ADTKD) and is characterized by enlarged kidneys, with a limited number of cysts, formed from all parts of the nephron. The extrarenal manifestations include early-onset diabetes (MODY5), genital malformation, hypomagnesemia, hyperuricemia, and elevated liver enzymes [12]. Renal cysts may also be detected in nephronophthisis (NPHP), an autosomal recessive tubulointerstitial disease, which accounts for about 5% of children with renal failure. In NPHP, kidneys are normally sized or small with bilateral hyper-echogenicity. Extrarenal manifestations of the disease may be helpful for making the right diagnosis [13].

ARPKD is genetically and phenotypically heterogeneous, and the severity of clinical symptoms is related to the type of mutation and the time at which symptoms first appear. And so, biallelic truncating mutations are associated with a severe phenotype, whereas carriers of two missense mutations have been observed to manifest a milder form of the disease. The most common mutation, accounting for about 15–20% of mutant alleles in *PKHD1*, is the T36M mutation (c.107C>T), called a “mutational hotspot”. Mutations in the *DZIP1L* gene are related to a moderate phenotype of disease [1]. Our patient is a heterogenous carrier of two missense mutations in the *PKHD1* gene inherited from parents and an additional missense mutation in the *PKD1* gene inherited from the mother. The obtained result regarding mutation in the *PKD1* gene is inconclusive due to the normal abdominal US image in the mother. However, the presence of the *PKD1* mutation is clinically not clear; dual *PKHD1-PKD1* mutations could have probably influenced the course of the disease and development of cysts in the liver.

Zingg-Schenk et al. reported a case of the coexistence of ARPKD and PCLD. Symptoms related to ARPKD were identified in a male newborn at the age of 2 weeks, while the presence of hepatic cysts was detected at the age of 9 years. In contrast to the case report we presented, the family history disclosed the presence of liver cysts in the boy’s mother at the age of 37 [14]. Genetic analysis showed the presence of heterogeneous mutations in *PKHD1* (maternally, c.1486C>T/p.Arg496X; paternally: c.5585C>T/p.Ser1862Leu) and a missense maternal mutation in exon 6 of the *PRKCSH* gene (c.416G>A; p.Arg139His), responsible for PCLD.

Liver cysts were also present in a 3-month-old boy with ARPKD, reported by Van Buren et al. The boy was diagnosed with biliary atresia due to jaundice and underwent the Kasai procedure. Abdominal US detected bilaterally enlarged cystic kidneys and echogenic cystic structures in the porta hepatis region and right hepatic lobe. Genetic tests revealed the presence of a heterogeneous variant in exon 62 of the *PKHD1* gene (c.11207T>A/p.I3736K; NM_138694.3; chr 6: 51513986). The variant was not identified in the genomes of the parents, although the family history revealed the presence of renal disease in three maternal relatives and a liver transplant at the age of 40 in the paternal grandfather [15].

The primary cilia is one of the most important organelles involved in the kidney’s and liver’s cyst formation. It is composed of a variety of signaling molecules, including polystin-1 (PC1)—protein encoded by the PKD1 gene; polycystic-2 (PC2)—protein encoded by the PKD2 gene; and fibrocystin (FPC)—protein encoded by the PKHD1 gene. Molecules PC1 and PC2 form a complex localized at the primary cilia, PC2 is an ion transporter that interacts with FCP, and FCP plays an important role in transporting gene products into the ciliary axoneme [5]. The function of proteins is to facilitate the transfer of information from the external environment to the cell, which is essential for maintaining normal renal tubule structure and function. The loss of either PC1 or PC2 is linked to reduced levels of intracellular calcium, elevated levels of cyclic AMP (cAMP), and activation of pathways involved in secretion and proliferation, thereby contributing to cystogenesis [16]. Loss of FPC expression does not affect the expression or localization of the PC1/PC2 complex. However, the hypothesis stating that PC1, PC2, and FPC interact with genetic pathways through, e.g., the proper regulation of Ca^2+^ flux is still possible [11,17]. Experimental studies have shown that mice and rats as carriers of both the *Pkhd1* and *Pkd1* mutations have a more rapid and more severe course of PKD. They have highlighted a synergistic relationship between *Pkhd1* and *Pkd1* knockout [4,18]. Murine models of ARPKD have shown dosage-dependent interaction between *Pkhd1* and *Pkd1* [3]. In PKD, cyst development occurred with reduced expression of *Pkd1*. The likelihood of cyst formation in the kidney increases when the level of functional PC1 or PC2 falls below a critical threshold, which is usually 20–30% (gene dosage effect/threshold) [16]. Experimental studies performed by Olson et al. showed that a reduction in PC1 in the context of FPC loss may cause rapidly progressive PKD [3].

Genetic analysis of individuals with isolated PCLD has identified *PKHD1* as the gene responsible for the disease. The majority of parents of children with ARPKD do not present any symptoms of the disease, although monoallelic loss of *PKHD1* has been observed in 10% of parents of ARPKD carriers who had asymptomatic liver cysts [10,17].

The underlying mechanisms of liver cyst formation in ARPKD are not well understood. Bile duct cysts may be present due to haploinsufficiency or because of somatic loss of the single normal allele [17]. Besse et al. reported that grown mice with deprived *Pkhd1* after normal liver development have a severe and fully penetrant cystic liver phenotype [18]. Mice with homozygous Pkhd1 mutations have kidney and liver cysts that were aggravated by reduced Pkd1 dosage [11,19]. Severe PKD models have shown that inadequate gene expression of *Pkhd1* or a single *Pkd1^RC^* does not intensify the phenotype, but severe disease results from homozygosity of either allele in a digenic manner [3]. The impact of modifying genetic or environmental factors on gene expression cannot be excluded, since not all *PKHD1* carriers develop liver cysts [11].

## 4. Conclusions

We present the case of a 5-year-old boy with a kidney US image typical of ARPKD and numerous large cysts in the liver not typical for this disease. Genetic testing revealed heterozygous missense mutations in the *PKHD1* gene and an additional new missense mutation in *PKD1*. However, the presence of the *PKD1* mutation is clinically not clear due to the normal abdominal US image in the mother; genetic findings seem to offer the most likely explanation for the unusual phenotype in our patient. This case report may contribute to the understanding of the phenotypic variability in ARPKD and the potential modifying role of mutations in other PKD-related genes. Comprehensive genetic panels are crucial for explaining atypical phenotypes and prognosis in patients with PKD.

## Data Availability

The original contributions presented in this study are included in the article. Further inquiries can be directed to the corresponding author.
